# Oral exposure to dibutyl phthalate exacerbates chronic lymphocytic thyroiditis through oxidative stress in female Wistar rats

**DOI:** 10.1038/s41598-017-15533-z

**Published:** 2017-11-13

**Authors:** Yang Wu, Jinquan Li, Biao Yan, Yuqing Zhu, Xudong Liu, Mingqing Chen, Dai Li, Ching-Chang Lee, Xu Yang, Ping Ma

**Affiliations:** 1 Laboratory of Environment- immunological and neurological Diseases, School of Basic Medical Sciences, Hubei University of Science and Technology, Xianning, 437100 China; 2 Laboratory of Environmental Biomedicine, College of Life Sciences, Central China Normal University, Wuhan, 430079 China; 3 Department of Environmental and Occupational Health, Medical College, National Cheng Kung University, Tainan, 70428 Taiwan

## Abstract

Chronic lymphocytic thyroiditis (CLT) is a common autoimmune disorder. The possible pathogenic role and mechanism of dibutyl phthalate (DBP) in CLT is still controversial. Experiments were conducted after 35-days of oral exposure to the three concentrations of DBP or saline, and three immunizations with thyroglobulin (TG). Healthy female Wistar rats were randomly divided into ten exposure groups (n = 8 each): (A) saline control, (B) 0.5 mg/kg/d DBP, (C) 5 mg/kg/d DBP, (D) 50 mg/kg/d DBP, (E) TG-immunized group, (F) TG- combined with 0.5 mg/kg/d DBP, (G) TG- combined with 5 mg/kg/d DBP, (H) TG- combined with 50 mg/kg/d DBP, (I) TG- combined with 50 mg/kg/d DBP plus 100 mg/kg/d vitamin C; (J) 100 mg/kg/d vitamin C. We showed that oral exposure DBP can aggravate CLT in rats. This deterioration was concomitant with increased thyroid auto antibodies, Th1/Th2 imbalance and Th17 immune response, activated pro-inflammatory and apoptosis pathways, and increased thyroid dysfunction in rats. Our results also suggested that DBP could promote oxidative damage. The study also found that vitamin C reduced the levels of oxidative stress and alleviated CLT. In short, the study showed that DBP exacerbated CLT through oxidative stress.

## Introduction

Chronic lymphocytic thyroiditis (CLT), also known as Hashimoto’s thyroiditis, is a common, prototypical, organ-specific autoimmune disorder. The incidence of CLT is estimated to affect 5% of the population, with women being more at risk than men^1,2^. CLT is characterized pathologically by infiltration of the thyroid mainly by T cells reactive to thyroid antigens, is characterized biochemically by the production of thyroid autoantibodies, and characterized clinically by abnormal thyroid function^3^. CLT is considered to be a T helper 1 (Th1) lymphocyte-mediated disease. Th1 lymphocytes in thyroid tissue may be responsible for enhanced interferon (IFN)-γ production, therefore creating an amplification feedback loop, initiating and perpetuating the autoimmune process^4^.

CLT is multifactorial, in that a genetic predisposition combines with environmental risk factors to promote the disease^5^. High levels of several chemical agents have been implicated in the incidence of CLT^6,7^. It is noteworthy that there are a wide variety of synthetic chemicals that have the ability to promote thyroid immune dysfunction in the host^8^.

Phthalates (PAEs) are a class of synthetic chemicals that are widely used in industrial and consumer products, including medical devices, food wrap, building materials, packaging, automotive parts and toys^9^. Humans are exposed to phthalates through ingestion, inhalation, and dermally throughout their lives, and this exposure can lead to health problems, including developmental and reproductive disruption^10^.

Phthalates are easily emitted since they are not tightly bound to the polymer matrix. Millions of pounds of phthalates are discharged into the environment every year, and individuals are exposed to phthalates in occupational and domestic environments^11^. In recent years, accumulating evidence from human studies has indicated that the thyroid is vulnerable to the endocrine disrupting effects of phthalates. In women, a significant negative association between the metabolite of dibutyl phthalate (DBP) and total thyroxine (T4) was found^12^, and other epidemiological data suggest that a reduction in thyroid hormone (TH) levels result from exposure to phthalates^13–15^. In addition, experimental results from toxicological studies also support these findings. In rats, DBP decreased Triiodothyronine (T3) and T4 in a dose-dependent manner^16^, and other studies have shown morphological changes in the thyroid after exposure to phthalates^17^. The prevalence of CLT has increased with the increase in environmental pollution, suggesting that certain environmental toxins, such as phthalates, may be implicated. However, the molecular mechanism behind phthalate-induced thyroid dysfunction still needs to be elucidated.

Reactive oxygen species (ROS)-induced oxidative stress could participate in the pathophysiology of various autoimmune diseases^18^. ROS are fundamental for the normal functioning of the thyroid follicular cell, and are physiologically necessary and intimately associated with thyroid hormone synthesis. However, an oversupply of ROS may be toxic^19^. Reports indicate that ROS have also been implicated in the pathogenesis of CLT, in both murine and human models^20^.

DBP is the primary plasticizer currently used in China, it has been listed as a priority environmental pollutant by the China National Environmental Monitoring Centre^21^. Experimental autoimmune thyroiditis (EAT) is an excellent model for CLT, EAT is induced with thyroglobulin (TG), a known thyroid auto-antigen that is common to both rats and humans^22^. Therefore, we used a female EAT rat model to study the suspected impact of DBP on CLT by investigating the levels of thyroid auto antibodies, tissue lesions cytokine, apoptosis factors, thyroid hormones and oxidative stress. We then evaluated the protective effect of vitamin C (VitC) on CLT and investigated its mechanism as an antioxidant.

## Results

### DBP exacerbating CLT induced by TG

**Figure 1:**
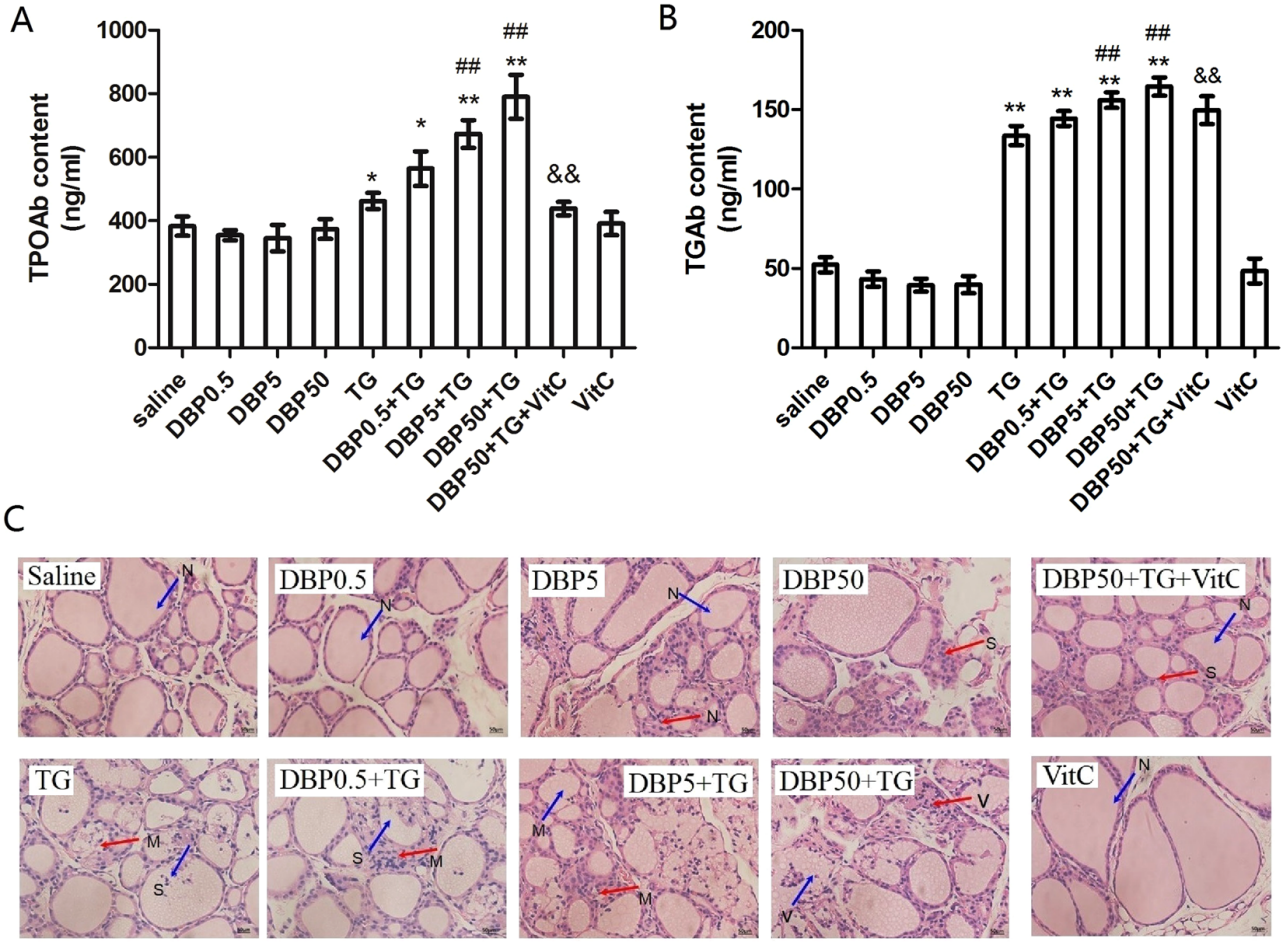
Thyroid auto antibody levels and histology. (**A**) TPOAb level in rat serum, F_DBP_ = 1.82 (p = 0.158), F_TG_ = 1212.4(p = 0.000), F_DBP*TG_ = 9.83 (p = 0.000). (**B**) TGAb level in rat serum, F_DBP_ = 6.40 (p = 0.001), F_TG_ = 90.31(p = 0.000), F_DBP*TG_ = 7.15(p = 0.001). (**C**) H&E staining showing infiltration of inflammatory cells (red arrow) and focal derangement (blue arrow). N: normal conditions; S: slight changes; M: moderate changes; V: severe changes. Magnification = ×40. ^##^p < 0.01, compared with the TG group. *p < 0.05, **p < 0.01, compared with the saline group; ^##^p < 0.01, compared with the TG group; ^&&^p < 0.01, compared with the DBP50 + TG group.

CLT is indicated by high levels of serum auto antibodies and thyroid lymphocytic infiltration. TPOAb and TGAb are the hallmark of chronic lymphocytic thyroiditis. Figure 1A,B show the levels of auto antibodies. Exposure in the DBP-only groups (DBP0.5, DBP5, DBP50) did not result in changes in serum TGAb and TPOAb levels (P_trend_ > 0.05), the TGAb and TPOAb levels for these groups were the same as the saline control group, including the DBP50 group. The serum TGAb and TPOAb levels of the TG-immunized groups (TG, DBP0.5 + TG, DBP5 + TG, DBP50 + TG) were significantly increased (P_trend_ < 0.0001). A significant increase (p < 0.01) was also observed in the TG combined with DBP groups (DBP5 + TG, DBP50 + TG), in comparison to the TG-alone group for both auto antibody levels. Compared with the DBP50 + TG group, the TPOAb and TGAb levels in the DBP50 + TG + VitC group were both significantly attenuated (p < 0.01).

As shown by H&E staining, typical pathological features of chronic lymphocytic thyroiditis can be seen in all the TG-immunized groups, in contrast to the non-immunized groups (Fig. 1C). The TG group rats exhibited focal derangement and lymphocytes were present, findings which are normally associated with chronic lymphocytic thyroiditis in humans. The follicles of rats in the DBP5 + TG groups exhibited moderate pathological changes, with lymphocyte infiltration. The follicles in the DBP50 + TG group showed a difference in size, diffuse follicle destruction and diffuse lymphocytic infiltration. In addition, slight changes were observed in the DBP50 group compared with the saline control group. These results indicate that exposure to DBP does not cause CLT but could exacerbate the CLT induced by TG.

### DBP aggravating the Th1/Th2 imbalance and Th17 immune response

**Figure 2:**
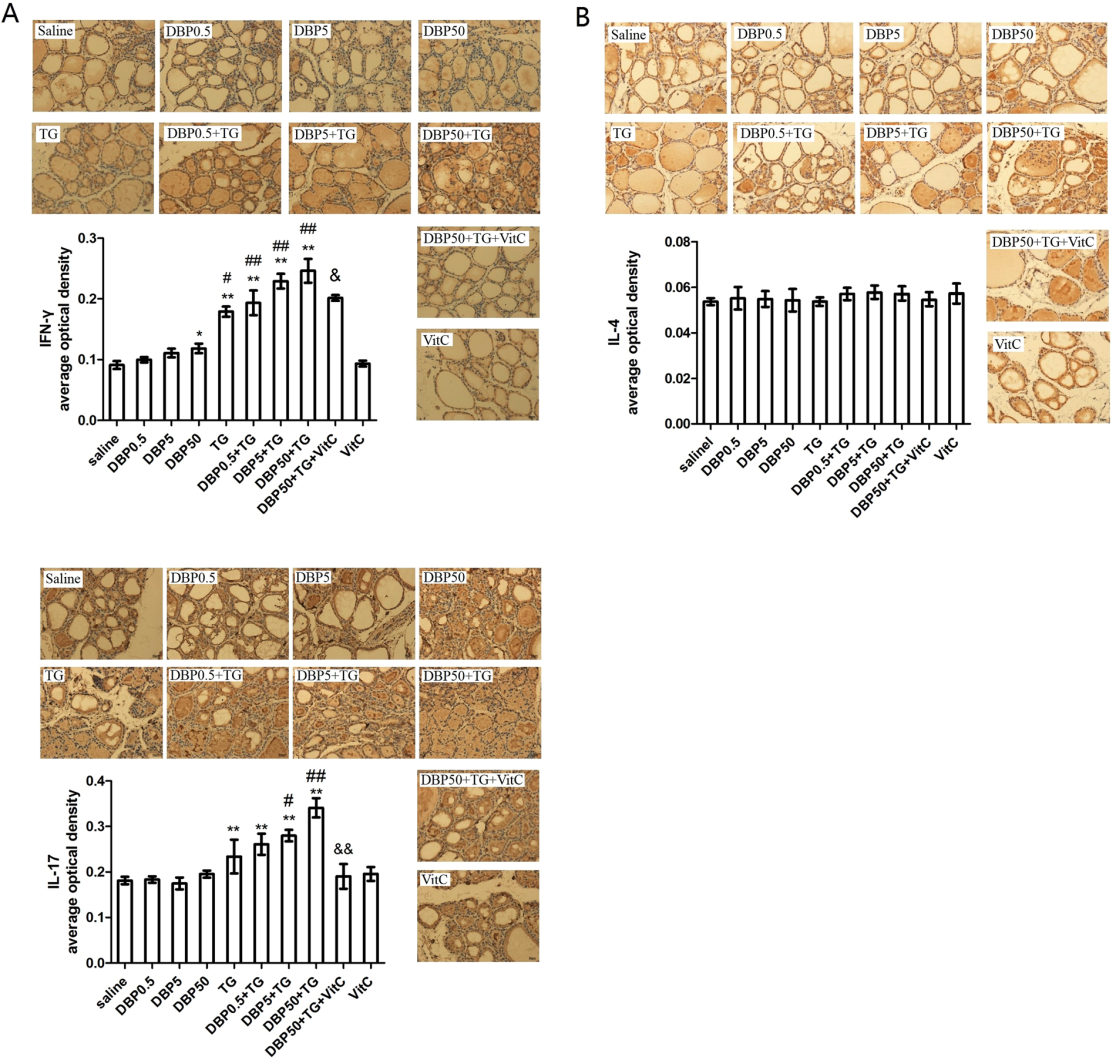
Th1/Th2 imbalance and Th17 immune response. (**A**) Immunohistochemistry and average optical density for IFN-γ, F_DBP_ = 5.596 (p = 0.002), F_TG_ = 142.67 (p = 0.000), F_DBP*TG_ = 1.114 (p = 0.351). (**B**) Immunohistochemistry and average optical density for IL-4, F_DBP_ = 0.537 (p = 0.659), F_TG_ = 1.419 (p = 0.24), F_DBP*TG_ = 0.168 (p = 0.917). (**C**) Immunohistochemistry and average optical density for IL-17 F_DBP_ = 6.966 (p = 0.001), F_TG_ = 93.46 (p = 0.000), F_DBP*TG_ = 4.136 (p = 0.011). Magnification = ×40. *p < 0.05, **p < 0.01, compared with the saline group; ^#^p < 0.05, ^##^p < 0.01, compared with the TG group; ^&^p < 0.05, ^&&^p < 0.01, compared with the DBP50 + TG group.

The typical Th1 cytokine IFN-γ, and Th2 cytokine IL-4 were detected in order to evaluate the lymphocyte cytokine response. The IFN-γ levels in the rats from the DBP-only, and the TG-immunized groups, showed significant dose-dependent increases (P_trend_ < 0.01). Moreover, the DBP50 group showed a significant increase compared with the saline control group (p < 0.05). The DBP5 + TG, DBP50 + TG groups showed very significant changes compared with the TG-only group (p < 0.01) (Fig. 2A). No significant change was observed in IL-4 levels among the experimental groups (Fig. 2B).

The Th17 associated cytokine, IL-17, was measured to reflect the Th17 immune response. Data indicate no significant dose-dependent induction in the DBP-only group (P_trend_ > 0.05), and there were no changes in the IL-17 levels seen among the other DBP groups (Fig. 2C). Nevertheless, DBP5 + TG, DBP50 + TG promoted the level of IL-17 in the TG-only group (p < 0.05, p < 0.01). In addition, significant dose dependent increases were observed in all TG-immunized groups (P_trend_ < 0.001) (Fig. 2C).

Treatment with Vitamin C dramatically reduced IFN-γ (p < 0.05) and IL-17 levels (p < 0.01) in the DBP50 + TG-treated rats (Fig. 2A,C). Treatment with VitC did not reduce IL-4 levels compared with DBP50 + TG group (p > 0.05) (Fig. 2B).

### DBP activation of pro-inflammatory mediator and apoptosis factor

**Figure 3:**
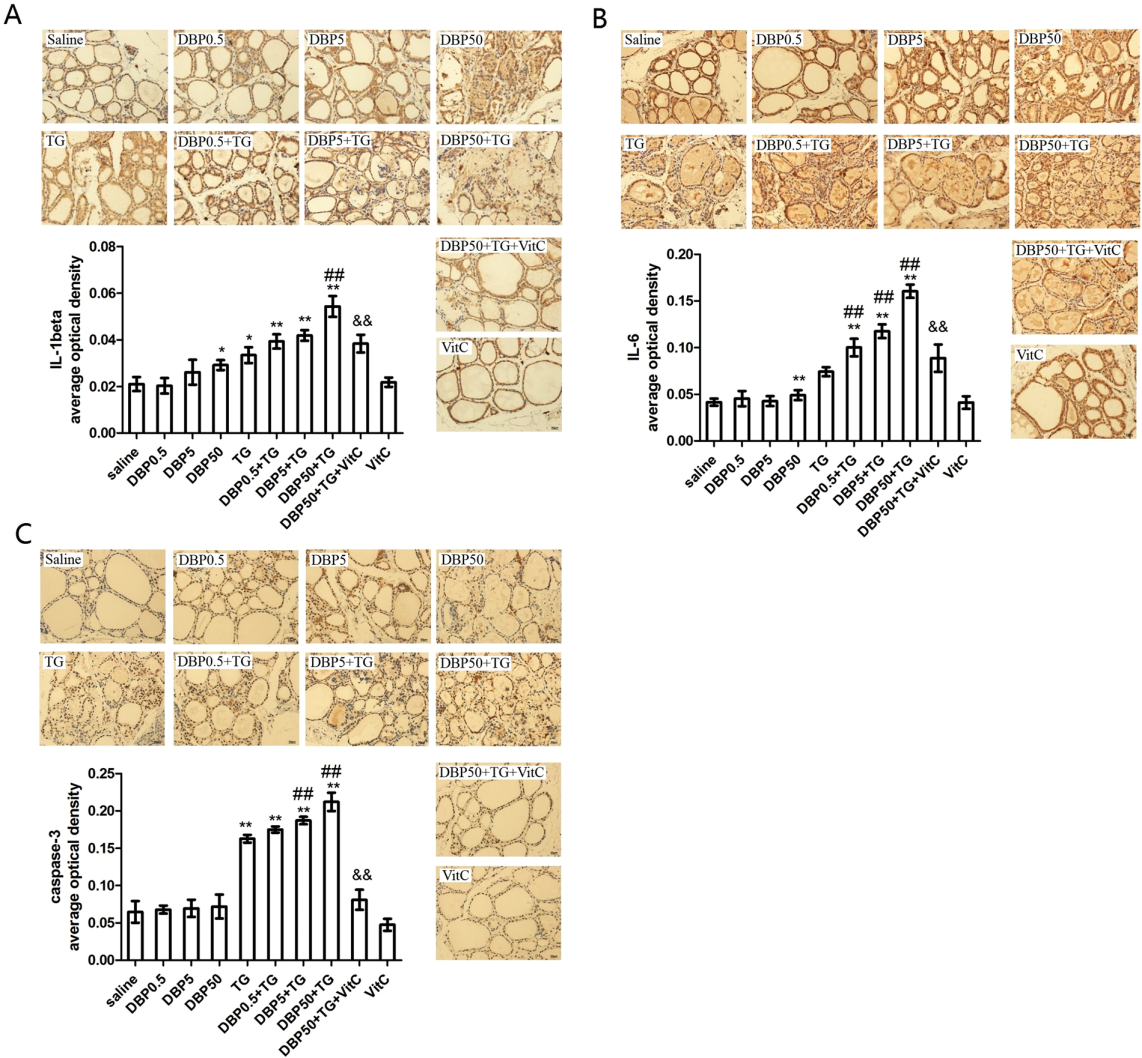
Pro-inflammatory and apoptosis factor. (**A**) Immunohistochemistry and average optical density for IL-1β, F_DBP_ = 6.52 (p = 0.001), F_TG_ = 52.47 (p = 0.000), F_DBP*TG_ = 1.196 (p = 0.32). (**B**) Immunohistochemistry and average optical density for IL-6, F_DBP_ = 35.6 (p = 0.000), F_TG_ = 420.4 (p = 0.000), F_DBP*TG_ = 25.89 (p = 0.000). (**C**) Immunohistochemistry and average optical density for caspase-3, F_DBP_ = 2.4 (p = 0.078), F_TG_ = 205.9 (p = 0.000), F_DBP*TG_ = 1.37 (p = 0.262). Magnification = ×40. *p < 0.05, **p < 0.01, compared with the saline group; ^##^p < 0.01, compared with the TG group; ^&&^p < 0.01, compared with the DBP50 + TG group.

IL-1β and IL-6, key mediators of the induced inflammatory response, are thought to contribute to many of the pathophysiological changes associated with inflammation. A significant increase in IL-6 levels was found in the DBP50 group (p < 0.05) and the DBP50 + TG group (p < 0.01) compared with the TG group. An increase in the DBP exposure dose was associated with an increase in IL-1β levels in the TG combined with DBP groups (P_trend_ < 0.001) (Fig. 3A). The IL-6 level was similarly increased in the DBP50 groups (p < 0.05), and strongly enhanced in the DBP0.5 + TG, DBP5 + TG, DBP50 + TG groups (p < 0.05). Significant dose dependent increases were observed in all TG-immunized groups (P_trend_ < 0.0001) (Fig. 3B).

Caspase-3 independent mechanisms are the most important mechanisms of apoptosis. A significant increase in the Caspase-3 level was observed in the DBP5 + TG and DBP50 + TG groups compared with the TG group (p < 0.01), whereas there was no significant change among the DBP-only groups (P_trend_ < 0.0001) (Fig. 3C). There is a significant decrease in IL-1β (p < 0.05), IL-6 (p < 0.01) and caspase-3 (p < 0.01) levels in the DBP50 + TG + VitC group, compared with the DBP50 + TG exposure group (Fig. 3A–C).

### DBP exacerbating thyroid dysfunction

**Figure 4:**
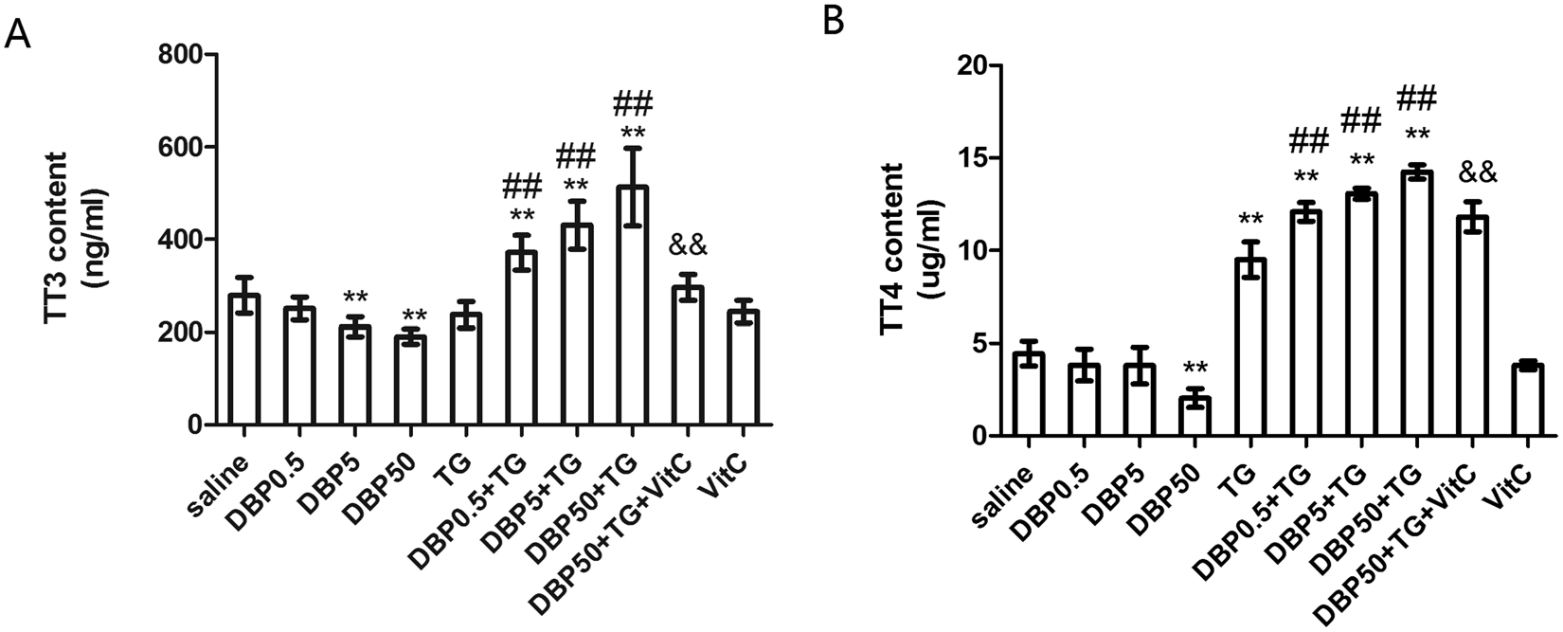
Thyroid function. (**A**) TT3 level in rat serum, F_DBP_ = 9.55 (p = 0.000), F_TG_ = 153.2 (p = 0.000), F_DBP*TG_ = 1.196 (p = 0.32). (**B**) TT4 level in rat serum, F_DBP_ = 6.52 (p = 0.001), F_TG_ = 52.47 (p = 0.000), F_DBP*TG_ = 37.74 (p = 0.000). **p < 0.01, compared with the saline group; ^##^p < 0.01, compared with the TG group; ^&&^p < 0.01, compared with the DBP50 + TG group.

The levels of thyroid function were investigated by measuring TT3 and TT4 content in the serum. When compared with the saline group, the DBP5 and DBP50 groups demonstrated a decrease in TT3 content (p < 0.05), while the DBP50 group also showed a decrease in TT4 content (p < 0.01). Both TT3 and TT4 levels indicate significant dose-dependent induction in the DBP-only group (TT3: P_trend_ < 0.0001; TT4: P_trend_ < 0.001) (Fig. 4A). However, compared with the TG group, the levels of TT3 and TT4 showed a significant increase in the DBP0.5 + TG, DBP5 + TG, DBP50 + TG groups (p < 0.05, p < 0.01, P_trend_ < 0.0001) (Fig. 4B). The TT3 and TT4 levels of the DBP50 + TG + VitC group decreased significantly compared with the DBP50 + TG group (p < 0.01).

### DBP aggravating oxidative stress

**Figure 5:**
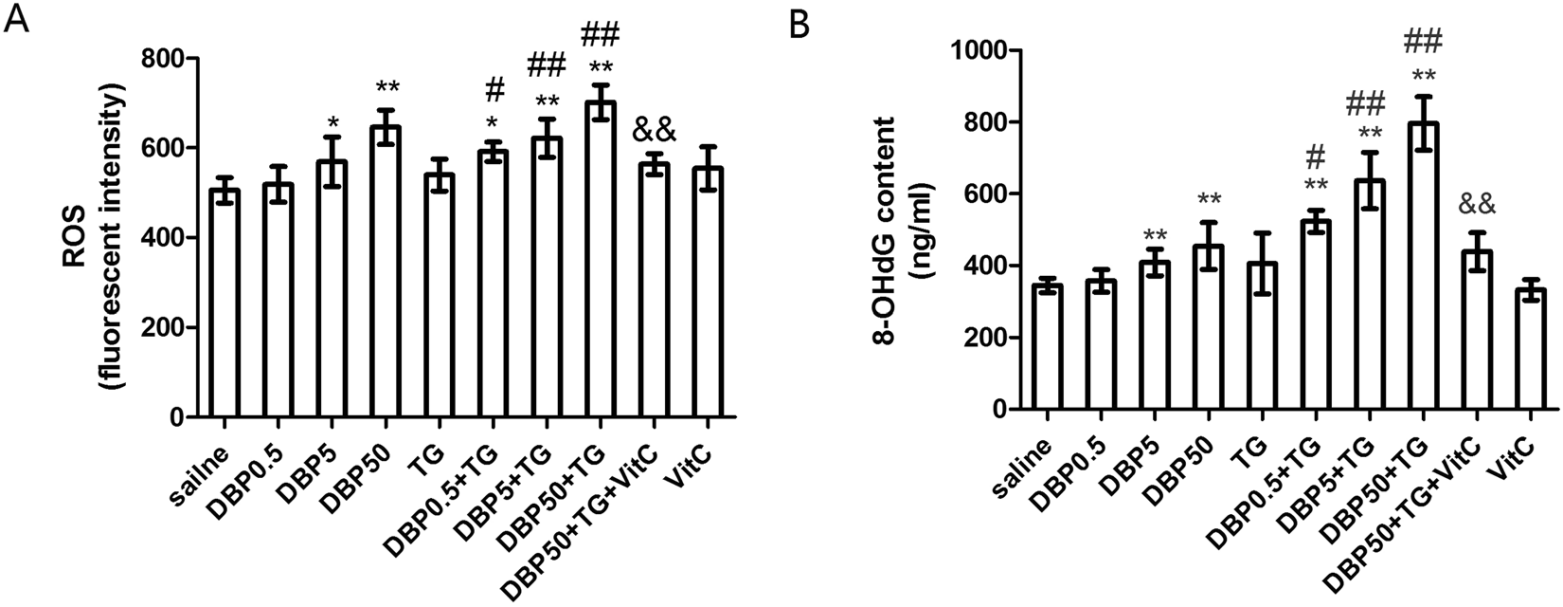
Oxidative stress. (**A**) ROS level in rat serum, F_DBP_ = 32.96 (p = 0.000), F_TG_ = 22.08 (p = 0.000), F_DBP*TG_ = 0.461 (p = 0.711). (**B**) 8-OHdG level in rat serum, F_DBP_ = 38.9 (p = 0.000), F_TG_ = 127.9 (p = 0.000), F_DBP*TG_ = 11.54 (p = 0.32). *p < 0.05, **p < 0.01, compared with the saline group; ^#^p < 0.05, ^##^p < 0.01, compared with the TG group; ^&&^p < 0.01, compared with the DBP50 + TG group.

ROS levels, the most important biomarker of oxidative stress, are shown in Fig. 5. The ROS levels in serum increased in a dose-dependent manner, with or without TG (P_trend_ < 0.0001). Moreover, the ROS concentration in the DBP5 and DBP50 groups was much higher than in the control group (p < 0.05, p < 0.01, P_trend_ < 0.0001). The same effect was observed in the DBP5 + TG and the DBP50 + TG groups as compared with the TG-only group (Fig. 5A).

The level of 8-OH-dG, a biomarker of DNA oxidative damage, was also measured (Fig. 5B). A significant increase in the 8-OHdG level was found in the DBP5 and DBP50 groups (p < 0.01, P_trend_ < 0.0001). Similar changes were noted in the DBP0.5 + TG, DBP5 + TG, DBP50 + TG groups as compared with the TG-only group (p < 0.05, p < 0.01, P_trend_ < 0.0001).

When DBP is administered together with VitC, the oxidative stress levels were reduced. There shows a significant decrease in the ROS level (p < 0.01) in the DBP50 + TG + VitC group when compared with the DBP50 + TG group (Fig. 5A). Compared with the DBP50 + TG group, the 8-OHdG level in the DBP50 + TG + VitC group also decreased significantly (p < 0.01) (Fig. 5B).

## Discussion

Although many studies suggest that DBP exposure may disrupt endocrine pathways in humans and animals, whether DBP is associated with a thyroid-disrupting effect is still controversial due to a lack of sufficient evidence regarding its toxicity. According to the tolerable daily intake (TDI) values for DBP (0.01 mg/kg/d) obtained by the Scientific Committee on Toxicity, Ecotoxicity and the Environment^23^, and taking into account specific medical conditions (i.e. 10 to 20 mg/kg/day during neonatal transfusion or maternal nutrition^24^) and the differences in drug tolerance between humans and rodents, the rats in this study were given a daily oral exposure dose of 0, 0.5, 5, or 50 mg/kg/d DBP solution according to which group they were assigned.

TG-mediated EAT in a rat has been characterized as an animal model of chronic lymphocytic thyroiditis, with mononuclear cell infiltration of the thyroid gland, and elevated levels of plasma auto antibodies. TPOAb and TGAb levels are associated with the degree of infiltration by lymphocytes, which may trigger the synthesis of auto antibodies^25^. Antibodies are produced mainly by lymphocytes infiltrating the thyroid gland. There is a high correlation between the concentration of anti-thyroid peroxidase antibodies and the degree of lymphocytic infiltration of the thyroid gland^26^. In the absence of TG, DBP exposure did not cause obvious histological or physiological changes in the rat, indicating that oral exposure to DBP in itself does not produce lymphocytic thyroiditis. Nevertheless, after 35 days of oral exposure, the 5 and 50 mg/kg/d DBP + TG exposure groups exhibited increased levels of TPOAb and TGAb in the serum; histological changes such as focal derangement and the presence of lymphocytes in the thyroid. These results show that oral exposure to DBP could exacerbate TG-induced CLT-like symptoms.

Imbalance in the Th1/Th2 immune response is a pathological basis of CLT and other immune diseases. Recruited T helper 1 (Th1) lymphocytes may be responsible for enhanced IFN-γ and TNF-α production in the thyroid tissue, while Th1 predominates in CLT^27^. In this study, the Th2 cytokine IL-4 levels were found to be not significantly different among the experimental groups, while levels of IFN-γ were found to be markedly higher in the TG-immunized rats exposed to DBP. This finding reflects that an acceleration in the imbalance of the Th1/Th2 cytokines contributes to the cause and evolution of CLT.

Th17 cells are considered to be potential targets for modern therapies for autoimmune conditions. These cells play a central role in the pathogenesis of the chronic inflammatory phenomenon and the tissue damage seen in CLT^28^. A report on the possible role of the Th17 cells indicated that they are necessary for the induction of an animal model of autoimmune thyroiditis^29^. Another study observed increased expression of the IL-17 gene in patients with CLT^30^. Pro-inflammatory cytokines such as IL-1β and IL-6 are generally associated with the stimulation of inflammation and autoimmunity, and may also stimulate production of T regulatory lymphocytes, inhibiting the development of autoimmune diseases. IL-1β was known to affect thyroid function by stimulating IL-6 secretion and modifying epithelium integrity. In cell cultures, IL-1 and IL-6 increase the proliferation of the thyroid follicular cells, but also have an inhibitory effect on thyrocytes during stimulation of these cells by the Thyroid-Stimulating Hormone (TSH)^31^. In this study, IL-17, IL-1β and IL-6 levels were found to be markedly increased in the TG-immunized rats exposed to DBP, suggesting that Th17 and pro-inflammatory cytokines play an important role in DBP exacerbation of chronic lymphocytic thyroiditis.

Release of apoptosis factors, and mitochondrial perturbation can induce apoptosis. Caspase-3 has a proteolytic effect and cleaves proteins at aspartic acid residues. Activated caspase-3 has an irreversible commitment towards cell death. Therefore, in apoptosis mechanisms, caspase-3 plays a pivotal role^32^. Our results showed that apoptosis was detected in the TG-immunized rats. The expression of caspase-3 increased in the thyroid tissue of these rats, inducing thyroid tissue damage.

Thyroid hormones are capable of influencing the function of cells in the body. In this study, we saw that after five weeks of oral exposure to DBP, the TT3 and TT4 levels decreased. These results are consistent with epidemiological research^13–15^. Another report indicated that DBP affected the T3-dependent activation of the TRβ gene in T3-induced metamorphosing tadpoles^33^. Our results showed that levels of TT3 and TT4 increased significantly in the TG combined with DBP groups, the unexpected opposite pattern shown by the DBP alone results. We speculate that DBP alone and DBP combined with TG may have different mechanisms for influencing the production of thyroid hormones. However, further studies are needed to confirm this hypothesis with assessment of various cytokines and chemotactic molecules in thyroid tissue.

ROS are key players in oxidative stress, and are the products of cellular metabolism, primarily in the mitochondria. ROS are produced at a low level during normal aerobic metabolism, and play an important role in the redox-dependent regulation of signal transduction processes^34^. ROS accumulation may lead to the formation of oxidative stress in tissues, resulting in further tissue oxidative damage. The most extensively studied DNA damage is the formation of 8-OH-dG. This oxidized DNA product is important because it is relatively easily formed, and is mutagenic and carcinogenic. It is a good biomarker of oxidative tissue damage^35^.

We observed that when TG sensitized rats were co-treated with DBP, the ROS and 8-OHdG content in the blood increased further, which demonstrated that oxidative stress was related to this CLT model. A study that used NOD.H2h4 mice suggested that increased autoimmunity in the thyroid triggered by iodine is the result of enhanced ICAM-1 expression on thyrocytes from the generation of excess ROS^20^. Moreover, VitC (ascorbic acid) scavenged the free radicals induced by co-exposure to DBP and TG, leading to a considerably lower level of intracellular ROS and 8-OHdG levels. These results also show that oxidative stress plays an important role in the DBP- exacerbated effect.

VitC is a very important and powerful antioxidant that works in aqueous environments of the body. Both physiological and pharmacological concentrations of VitC are able to protect against free radical harm, which has been confirmed by *in vitro* and *in vivo* studies^36^. In this paper, we reported that VitC reduced CLT, Th1/Th2 imbalance, Th17 immune response, pro-inflammatory and apoptosis factor, and thyroid dysfunction in rats. Thus, the ROS pathway could be one possible mechanism for DBP aggravation in TG-induced CLT. DBP, in combination with TG, triggers production of ROS, leading to the initiation of the sensitizing process and deterioration of CLT.

This study shows that long-term oral DBP exposure can exacerbate CLT in rats. Moreover, long-term oral exposure to environmental toxins such as phthalates may endow an autoimmunity in humans or animals. This deterioration was mediated by oxidative stress, concomitant with exacerbating the Th1/Th2 imbalance and Th17 immune response, activation of pro-inflammatory and apoptosis pathways, with an enhancement in thyroid dysfunction. These findings significantly improve our understanding of the cellular mechanisms driving the aggravation effect of DBP and provide new explanations for the increased prevalence of autoimmune diseases.

## Materials and Methods

### Ethics statement

All experimental procedures were approved by the Office of Scientific Research Management of Hubei University of Science and Technology (Xianning, China) with a Certificate on Application for the Use of Animals dated 26 February 2016 (approval ID: HBUST-IACUC- 2016-001). All experiments in this study were performed in accordance with the approved guidelines and regulations.

### Experimental animals

Specified pathogen-free (SPF) class female Wistar rats (6-7 weeks old) were purchased from Hubei Experimental Animal Center (Wuhan, China). We used female rats in our study because women have a much greater risk of CLT than do men^1,2^. All rats were housed under SPF conditions at 20–25 °C with 50–70% humidity and a 12 h light/dark cycle. A commercial diet and filtered water were provided *ad libitum*. Rats were quarantined for ≥7 days prior to the study. Eight rats were used in each group in order to minimize the number of experimental animals needed while still ensuring statistical validity.

### Main reagents and kits

DBP (>99%), thyroglobulin (TG), complete Freund’s adjuvant (CFA), incomplete Freund’s adjuvant (IFA), vitamin C (VitC), formalin solution (10%), Tween-80 and pentobarbital sodium were obtained from Sigma-Aldrich (St. Louis, MO, USA). Rat enzyme-linked immunosorbent assay (ELISA) kits for TT4, TT3, TPO-antibodies (TPOAb), TG-antibodies (TGAb) and 8-hydroxy-2-deoxyguanosine (8-OHdG) were purchased from BlueGene Biotech (Shanghai, China), goat-anti-rabbit immunoglobulin (lg) G-antibody, rabbit lgG peroxidase conjugated streptavidin-biotin complex (SABC-POD) kit and diaminobenzidine (DAB) kit were obtained from Boster Bio-engineering (Wuhan, China). 2,7-dichlorodihydro -fluorescein diacetate (DCFH-DA) was purchased from Beyotime Biotech (Shanghai, China). All other chemicals were of analytical grade and were purchased from Sinopharm Chemical Reagent Co. (Shanghai, China).

### Experimental protocol

**Figure 6:**
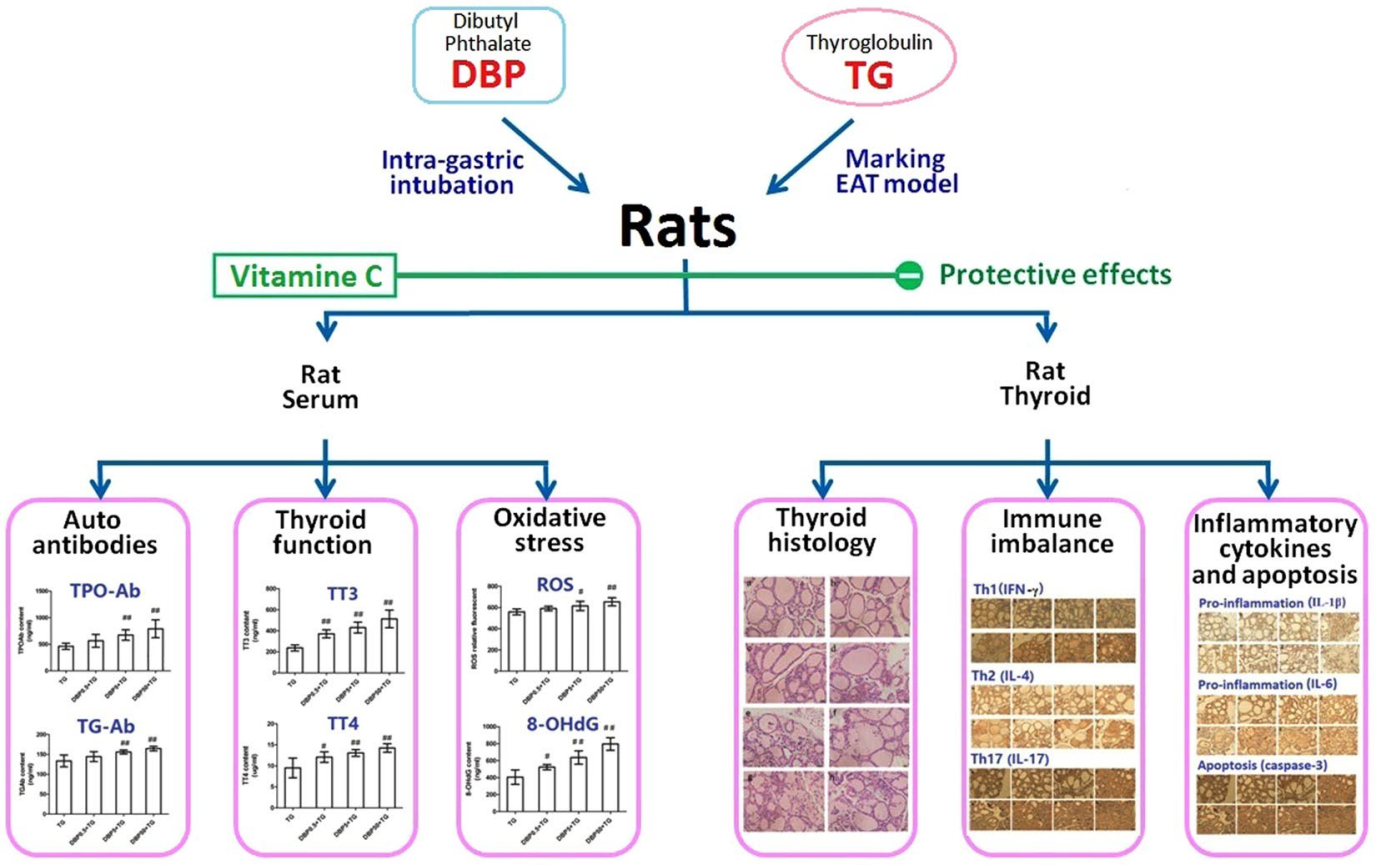
The study road map of the experiment.

The rats were divided randomly into ten groups of eight rats each. The groups were treated as follows: Group A: Saline oral exposure (saline); Group B: 0.5 mg/kg/d DBP oral exposure (DBP 0.5); Group C: 5 mg/kg/d DBP oral exposure (DBP 5); Group D: 50 mg/kg/d DBP oral exposure (DBP 50); Group E: Saline oral exposure combined with TG (TG); Group F: 0.5 mg/ (kg.d) DBP oral exposure combined with TG (DBP0.5 + TG); Group G: 5 mg/(kg.d) DBP oral exposure combined with TG (DBP5 + TG); Group H: 50 mg/(kg.d) DBP oral exposure combined with TG (DBP50 + TG); Group I: 50 mg/(kg.d) DBP oral exposure combined with TG plus 100 mg/(kg.d) vitamin C (DBP50 + TG + VitC); Group J: 100 mg/kg/day vitamin C (VitC). Groups A-I were given a daily gavage of saline or one of the three different concentrations of DBP. DBP was dissolved in Tween 80 (DBP: Tween-80 is 1:1 in w/w) together with a saline solution. The concentration of Tween-80 was previously demonstrated to be pharmacologically inert in *in vivo* experiments^37^. Animals received daily DBP intragastric intubations via a metal gastric tube for 35 consecutive days. TG (100 μg) in 400 μL of saline was emulsified with an equal volume of CFA, and the emulsion was then injected subcutaneously at multiple sites on the neck and back of rats on day 8. On the 15th and 29th days, the rats were boosted with an additional TG (100 μg) in 400 μL of saline emulsified an equal volume of IFA. Groups I and J received a daily dose of VitC as an anti-oxidant via intragastric administration. The protocols are shown in Fig. 6.

### Quantitative analyses of thyroid auto antibodies and hormones

The rats were anesthetized intraperitoneally with pentobarbital sodium (100 mg/kg bw). Serum samples were then extracted from heart blood by centrifugation (3000 rpm at 4 °C for 15 min) and stored at −70 °C. The serum levels of TPOAb, TGAb, TT4 and TT3 were measured using the ELISA kits according to the manufacturer’s instructions. Concentrations were determined in duplicate for each sample. The sensitivities of the kits were 0.1 μg/mL for TT4, 1.0 ng/mL for TT3, TPOAb and TGAb.

### Thyroid histological examination

Rats were killed by cervical dislocation. The thyroid glands were collected and fixed in 10% formalin solution for 24 h at room temperature, following which the distal pieces were embedded in paraffin, sectioned into 5 μm slices for H&E staining. Each section was observed using a DP73 microscope (Olympus, Tokyo, Japan). Tissue sections were examined qualitatively by two experienced pathologists in a blinded fashion.

### Thyroid immunohistochemistry assay

Sections of thyroid tissue were incubated with 3% hydrogen peroxide (H_2_O_2_) and blocked by appropriate normal serum of endogenous peroxides_._ The sections were then boiled in sodium citrate (0.01 mol/L, pH 6.0) for antigen retrieval to unmask the antigen epitopes, permeabilized with 0.2% Triton X-100 for 10 min, and blocked with 5% bovine serum albumin (BSA) in phosphate buffer saline (PBS, PH = 7.4) for 30 min at room temperature. Immunohistochemical detection of IFN-γ, IL-4, IL-17, IL-6, IL-1β and caspase-3 was performed using primary antibodies: rat anti-IL-6 (Abcam, Cambridge, USA), rat anti-IL-1β and rat anti-Caspase-3 (Ruiying Biotech, Suzhou, China), rat-IL-4, rat anti-IFN-γ and rat anti-IL-17-antibody (Boster Bio-engineering). Sections were incubated with diluted primary antibodies overnight at 4 °C. Slides were washed with PBS, incubated with secondary antibodies for 30 min at 37 °C and detected with a rabbit IgG peroxidase conjugated streptavidin-biotin complex (SABC-POD) kit, followed by incubation with a diaminobenzidine (DAB) kit. Immunostained sections were viewed under a DP73 microscope. The staining intensity was determined as an average optical density using Image-Pro Plus 6.0 software (Media Cybernetics, Bethesda, MD, USA). A non-stained region was selected and set as the background. All tissue sections were examined qualitatively by two experienced pathologists in a blinded fashion.

### Detection of ROS and 8-OHdG content

Levels of ROS in the serum were determined based on the reactions between ROS and the byproducts of 2,7-dichlorofluorescein (DCFH)-DA^38^. After transfer into cells, DCFH-DA is cleaved to form DCFH, which in turn is transformed into highly fluorescent DCF upon reaction with ROS. DCF was quantified in each sample using a fluorescence monitor (FLx 800 Multi-Detection Microplate Reader, BioTek Instruments, Wisnooski, VT, USA). Initially, 2 μL of sample solution was removed to a test tube, and 198 μL of phosphate-buffered saline (PBS) at pH 7.5 was added. Then, 100 μL of the sample solution was removed to a 96-well microplate, and 100 μL of DCFH-DA fluorescent dye was added, diluted 1000-fold by PBS (pH = 7.5). The level of ROS in the supernatant was detected using a fluorescent microplate spectrophotometer at an excitation wavelength of 485 nm and an emission wavelength of 525 nm.

The 8-OH-dG concentration in the serum was measured using an ELISA kit, according to the manufacturer’s instructions. Concentrations were determined in duplicate for each sample. The sensitivity of the kit is 0.1ng/mL.

### Statistical analyses

All data are the mean ± standard error of the mean. Statistical graphs were generated using GraphPad Prism 5.02 (San Diego, CA, USA). A two-way analysis of variance (ANOVA) combined with an LSD t-test was used to determine the significance of differences between the groups, p < 0.05 was considered significant and p < 0.01 was considered extremely significant. The dose-response relationship was tested by linear regression, P_trend_ < 0.05 was considered statistically significant. Data analyses were carried out using SPSS ver18 (SPSS, Chicago, IL, USA).

### Data availability

The datasets generated and/or analyzed during the current study are available from the corresponding author upon reasonable request. All data generated or analyzed during this study are included in this published article.

## Electronic supplementary material

Dataset 1

## References

[1] McLeod DSA, Cooper DS (2012). The incidence and prevalence of thyroid autoimmunity. Endocrine.

[2] Pyzik, A., Grywalska, E., Matyjaszek-Matuszek, B. & Roliński, J. Immune disorders in Hashimoto’s thyroiditis: what do we know so far? *J Immunol Res*. 979167 (2015). 10.1155/2015/979167 PMC4426893 26000316

[3] Antonelli A, Ferrari SM, Corrado A, Domenicantonio A, Fallahi P (2015). Autoimmune thyroid disorders. Autoimmun Rev..

[4] Wiersinga WM (2014). Thyroid Autoimmunity. Endocr Dev..

[5] Tomer Y, Huber A (2009). The etiology of autoimmune thyroid disease: A story of genes and environment. J Autoimmun..

[6] Burek CL, Talor MV (2009). Environmental triggers of autoimmune thyroiditis. J Autoimmun..

[7] Duntas LH (2011). Environmental factors and thyroid autoimmunity. Annales d’Endocrinologie.

[8] Brent GA (2010). Environmental Exposures and Autoimmune. Thyroid Disease. Thyroid.

[9] Lin CY (2017). Positive Association between Urinary Concentration of Phthalate Metabolites and Oxidation of DNA and Lipid in Adolescents and Young Adults. Sci. Rep..

[10] Swan SH (2008). Environmental phthalate exposure in relation to reproductive outcomes and other health endpoints in human. Environ Res..

[11] Latini G (2005). Monitoring phthalate exposure in humans. Clinica Chimica Acta..

[12] Huang PC, Kuo PL, Guo YL, Liao PC, Lee CC (2008). Associations between urinary phthalate monoesters and thyroid hormones in pregnant women. Hum Reprod..

[13] Meeker JD, Calafat AM, Hauser R (2007). Di (2-ethylhexyl) phthalate metabolites may alter thyroid hormone levels in men. Environ Health Persp..

[14] Boas M (2010). Childhood exposure to Phthalates: Associations with Thyroid Function, Insulin-like Growth Factor I, and Growth. Environ Health Persp..

[15] Wu MT (2013). Intake of Phthalate-Tainted Foods Alters Thyroid Functions in Taiwanese Children. PLoS ONE.

[16] O’Connor JC, Frame SR, Ladics GS (2002). Evaluation of a 15-day screening assay using intact male rats for identifying antiandrogens. Toxicol Sci..

[17] Howarth JA, Price SC, Dobrota M (2001). Effects on male rats of di-(2-ethylhexyl) phthalate and di-n-hexylphthalate administered alone or in combination. Toxicol Lett..

[18] Kurien BT, Hensley K, Bachmann M, Scofield RH (2006). Oxidatively modified autoantigens in autoimmune diseases. Free Radical Bio Med..

[19] Di Dalmazi G, Hirshberg J, Lyle D, Freij JB, Caturegli P (2012). Reactive oxygen species in organ-specific autoimmunity. Autoimmun Highlights.

[20] Burek CL, Rose NR (2008). Autoimmune thyroiditis and ROS. Autoimmun Rev..

[21] Wang Z (2016). The microbiome and functions of black soils are altered by dibutyl phthalate contamination. Appl Soil Ecol..

[22] Farine JC (1997). Animal models in autoimmune disease in immunotoxicity assessment. Toxicology..

[23] Scientific Committee on Health and Environmental Risks (SCHER) of the European Commission. 5. What daily exposure levels to phthalates are considered safe? Phthalates in school supplies http://ec.europa.eu/health/scientific_committees/opinions_layman/en/phthalates-school-supplies/index.htm# (2008).

[24] Loff S (2000). Polyvinylchloride infusion lines expose infants to large amounts of toxic plasticizers. J Pediatr Surg..

[25] Cui, S., Yu, J. & Shoujun, L. Iodine Intake Increases IP-10 Expression in the Serum and Thyroids of Rats with Experimental Autoimmune Thyroiditis. Int J Endocrinol. 581069 (2014). 10.1155/2014/581069 PMC3953660 24707288

[26] Cogni G, Chitgto L (2013). An overview of the pathogenesis of thyroid autoimmunity. Hormones.

[27] Weetman AP (2004). Cellular immune responses in autoimmune thyroid disease. Clin Endocrinol..

[28] Roberto G, Marazuela MT (2016). regulatory (Treg) and T helper 17 (Th17) lymphocytes in thyroid autoimmunity. Endocrine.

[29] Horie I (2009). T helper type 17 immune response plays an indispensable role for development of iodine-induced autoimmune thyroiditis in nonobese diabetic-H2h4 mice. Endocrinology.

[30] Qin Q (2012). The increased but non-predominant expression of Th17- and Th1-specific cytokines in Hashimoto´s thyroiditis but not in Graves´ disease. Braz J Med Biol Res..

[31] Zhao R, Zhou H, Su S (2013). A critical role for interleukin-1β in the progression of autoimmune diseases. Int Immunopharmaco..

[32] Wang S, Baker JR (2007). The Role of Apoptosis in. Thyroid Autoimmunity. Thyroid.

[33] Wenzel A, Franz C, Breous E, Loos U (2005). Modulation of iodide uptake by dialkyl phthalate plasticisers in frtl-5 rat thyroid follicular cells. Mol Cell Endocrino..

[34] Finkel T, Holbrook NJ (2000). Oxidants, oxidative stress and the biology of ageing. Nature.

[35] Valko M, Rhodes CJ, Moncol J, Izakovic M, Mazur M (2006). Free radicals, metals and antioxidants in oxidative stress- induced cancer. Chem-Biol Interact..

[36] Valko M (2007). Free radicals and antioxidants in normal physiological functions and human disease. Int J Biochem Cell Biol..

[37] Dimitrov M (2011). Acute toxicity, antidepressive and mao inhibitory activity of mangiferin isolated from hypericum aucheri. J. Biotechnol. Biotechnol. Eq..

[38] Ma P (2015). Cognitive defcits and anxiety induced by diisononyl phthalate in mice and the neuroprotective effects of melatonin. Sci. Rep..

