# Supplementary material for: Oral exposure to dibutyl phthalate exacerbates chronic lymphocytic thyroiditis through oxidative stress in female Wistar rats
Source: Sci Rep. 2017 Nov 13;7:15469. doi: 10.1038/s41598-017-15533-z (PMC5684247; doi:10.1038/s41598-017-15533-z)
Supplement: Supplementary file 1 — Dataset 1 [file 41598_2017_15533_MOESM1_ESM.doc]

**Results of the Two-way ANOVA**

**Title Page**

Oral exposure to dibutyl phthalate exacerbates chronic lymphocytic thyroiditis through oxidative stress in female Wistar rats

Yang Wu^1#^, Jinquan Li^2#^, Biao Yan^1^, Yuqing Zhu^2^, Xudong Liu^2^, Mingqing Chen^2^, Dai Li^1^, Ching-Chang Lee^3^*, Xu Yang^1, 2^*, Ping Ma^1^*,

1. Laboratory of Environment- immunological and neurological Diseases, School of Basic Medical Sciences, Hubei University of Science and Technology, Xianning 437100, China

2. Laboratory of Environmental Biomedicine, College of Life Sciences, Central China Normal University, Wuhan 430079, China

3. Department of Environmental and Occupational Health, Medical College, National Cheng Kung University, Tainan 70428, Taiwan

# These authors contributed equally to this work

* Corresponding authors:

Ping Ma, School of Basic Medical Science, Hubei University of Science and Technology, Xianning 437100, China. (Tel): +86-13886509837; (Email): mping68@126.com

Xu Yang, College of Life Sciences, Central China Normal University, Wuhan 430079, China. (Tel) 86-13871361954; (Email): yangxu@mail.ccnu.edu.cn

Ching-Chang Lee, Department of Environmental and Occupational Health, Medical College, National Cheng Kung University, Tainan, Taiwan: cclee@mail.ncku.edu.tw

**Results of the Two-way ANOVA**

**Summary of the P value**

|  | DBP | TG | DBP * TG |
| --- | --- | --- | --- |
| TGAb | 0.158 (NS*) | 0.000 | 0.000 |
| TPOAb | 0.001 | 0.000 | 0.000 |
| IFN-y | 0.002 | 0.000 | 0.351 (NS) |
| IL-4 | 0.659 (NS) | 0.240 (NS) | 0.917 (NS) |
| IL-17 | 0.001 | 0.000 | 0.011 |
| IL-6 | 0.000 | 0.000 | 0.000 |
| IL-1β | 0.001 | 0.000 | 0.320 |
| Caspases-3 | 0.078 (NS) | 0.000 | 0.262 (NS) |
| TT3 | 0.000 | 0.000 | 0.000 |
| TT4 | 0.000 | 0.000 | 0.000 |
| 8-OHdG | 0.000 | 0.000 | 0.000 |
| ROS | 0.000 | 0.000 | 0.711 (NS) |

*: No significant difference

**Figure 1：**

| **Tests of Between-Subjects Effects** | | | | | |
| --- | --- | --- | --- | --- | --- |
| Dependent Variable: TGAb | | | | | |
| Source | Type III Sum of Squares | df | Mean Square | F | Sig. |
| Corrected Model | 155988.862^a^ | 7 | 22284.123 | 182.500 | .000 |
| Intercept | 493769.104 | 1 | 493769.104 | 4043.816 | .000 |
| DBP | 664.882 | 3 | 221.627 | 1.815 | .158 |
| TG | 148043.666 | 1 | 148043.666 | 1212.432 | .000 |
| DBP * TG | 3601.214 | 3 | 1200.405 | 9.831 | .000 |
| Error | 5616.818 | 46 | 122.105 |  |  |
| Total | 694946.668 | 54 |  |  |  |
| Corrected Total | 161605.680 | 53 |  |  |  |
| a. R Squared = .965 (Adjusted R Squared = .960) | | | | | |

| **Tests of Between-Subjects Effects** | | | | | |
| --- | --- | --- | --- | --- | --- |
| Dependent Variable: TPOAb | | | | | |
| Source | Type III Sum of Squares | df | Mean Square | F | Sig. |
| Corrected Model | 1.103E6 | 7 | 157616.077 | 20.125 | .000 |
| Intercept | 1.033E7 | 1 | 1.033E7 | 1318.495 | .000 |
| DBP | 150455.874 | 3 | 50151.958 | 6.404 | .001 |
| TG | 707306.965 | 1 | 707306.965 | 90.313 | .000 |
| DBP * TG | 168069.214 | 3 | 56023.071 | 7.153 | .001 |
| Error | 274111.585 | 35 | 7831.760 |  |  |
| Total | 1.185E7 | 43 |  |  |  |
| Corrected Total | 1377424.123 | 42 |  |  |  |
| a. R Squared = .801 (Adjusted R Squared = .761) | | | | | |

**Figure 2：**

| **Tests of Between-Subjects Effects** | | | | | |
| --- | --- | --- | --- | --- | --- |
| Dependent Variable: IFN-γ | | | | | |
| Source | Type III Sum of Squares | df | Mean Square | F | Sig. |
| Corrected Model | .197^a^ | 7 | .028 | 22.815 | .000 |
| Intercept | 1.547 | 1 | 1.547 | 1255.196 | .000 |
| DBP | .021 | 3 | .007 | 5.596 | .002 |
| TG | .176 | 1 | .176 | 142.670 | .000 |
| DBP * TG | .004 | 3 | .001 | 1.114 | .351 |
| Error | .067 | 54 | .001 |  |  |
| Total | 1.839 | 62 |  |  |  |
| Corrected Total | .263 | 61 |  |  |  |
| a. R Squared = .747 (Adjusted R Squared = .715) | | | | | |

| **Tests of Between-Subjects Effects** | | | | | |
| --- | --- | --- | --- | --- | --- |
| Dependent Variable:IL-4 | | | | | |
| Source | Type III Sum of Squares | df | Mean Square | F | Sig. |
| Corrected Model | .000^a^ | 7 | 1.570E-5 | .506 | .824 |
| Intercept | .150 | 1 | .150 | 4845.413 | .000 |
| DBP | 4.999E-5 | 3 | 1.666E-5 | .537 | .659 |
| TG | 4.400E-5 | 1 | 4.400E-5 | 1.419 | .240 |
| DBP * TG | 1.565E-5 | 3 | 5.216E-6 | .168 | .917 |
| Error | .001 | 41 | 3.102E-5 |  |  |
| Total | .152 | 49 |  |  |  |
| Corrected Total | .001 | 48 |  |  |  |
| a. R Squared = .080 (Adjusted R Squared = -.078) | | | | | |

| **Tests of Between-Subjects Effects** | | | | | |
| --- | --- | --- | --- | --- | --- |
| Dependent Variable:IL-17 | | | | | |
| Source | Type III Sum of Squares | df | Mean Square | F | Sig. |
| Corrected Model | .166^a^ | 7 | .024 | 18.603 | .000 |
| Intercept | 2.821 | 1 | 2.821 | 2207.987 | .000 |
| DBP | .027 | 3 | .009 | 6.966 | .001 |
| TG | .119 | 1 | .119 | 93.461 | .000 |
| DBP * TG | .016 | 3 | .005 | 4.136 | .011 |
| Error | .057 | 45 | .001 |  |  |
| Total | 3.089 | 53 |  |  |  |
| Corrected Total | .224 | 52 |  |  |  |
| a. R Squared = .743 (Adjusted R Squared = .703) | | | | | |

**Figure 3：**

| **Tests of Between-Subjects Effects** | | | | | |
| --- | --- | --- | --- | --- | --- |
| Dependent Variable:IL_1beta | | | | | |
| Source | Type III Sum of Squares | df | Mean Square | F | Sig. |
| Corrected Model | .008^a^ | 7 | .001 | 11.847 | .000 |
| Intercept | .065 | 1 | .065 | 707.982 | .000 |
| DBP | .002 | 3 | .001 | 6.521 | .001 |
| TG | .005 | 1 | .005 | 52.465 | .000 |
| DBP * TG | .000 | 3 | .000 | 1.196 | .320 |
| Error | .005 | 52 | 9.159E-5 |  |  |
| Total | .082 | 60 |  |  |  |
| Corrected Total | .012 | 59 |  |  |  |
| a. R Squared = .615 (Adjusted R Squared = .563) | | | | | |

| **Tests of Between-Subjects Effects** | | | | | |
| --- | --- | --- | --- | --- | --- |
| Dependent Variable:IL_6 | | | | | |
| Source | Type III Sum of Squares | df | Mean Square | F | Sig. |
| Corrected Model | .094^a^ | 7 | .013 | 86.678 | .000 |
| Intercept | .345 | 1 | .345 | 2235.804 | .000 |
| DBP | .016 | 3 | .005 | 35.596 | .000 |
| TG | .065 | 1 | .065 | 420.443 | .000 |
| DBP * TG | .012 | 3 | .004 | 25.899 | .000 |
| Error | .007 | 48 | .000 |  |  |
| Total | .429 | 56 |  |  |  |
| Corrected Total | .101 | 55 |  |  |  |
| a. R Squared = .927 (Adjusted R Squared = .916) | | | | | |

| **Tests of Between-Subjects Effects** | | | | | |
| --- | --- | --- | --- | --- | --- |
| Dependent Variable:caspases_3 | | | | | |
| Source | Type III Sum of Squares | df | Mean Square | F | Sig. |
| Corrected Model | .213^a^ | 7 | .030 | 31.693 | .000 |
| Intercept | .941 | 1 | .941 | 981.801 | .000 |
| DBP | .007 | 3 | .002 | 2.400 | .078 |
| TG | .197 | 1 | .197 | 205.837 | .000 |
| DBP * TG | .004 | 3 | .001 | 1.370 | .262 |
| Error | .050 | 52 | .001 |  |  |
| Total | 1.198 | 60 |  |  |  |
| Corrected Total | .262 | 59 |  |  |  |
| a. R Squared = .810 (Adjusted R Squared = .785) | | | | | |

**Figure 4**：

| **Tests of Between-Subjects Effects** | | | | | |
| --- | --- | --- | --- | --- | --- |
| Dependent Variable:TT3 | | | | | |
| Source | Type III Sum of Squares | df | Mean Square | F | Sig. |
| Corrected Model | 484549.302^a^ | 7 | 69221.329 | 39.738 | .000 |
| Intercept | 4279816.162 | 1 | 4279816.162 | 2456.928 | .000 |
| DBP | 49882.106 | 3 | 16627.369 | 9.545 | .000 |
| TG | 266750.723 | 1 | 266750.723 | 153.134 | .000 |
| DBP * TG | 197195.619 | 3 | 65731.873 | 37.735 | .000 |
| Error | 64451.705 | 37 | 1741.938 |  |  |
| Total | 4791674.637 | 45 |  |  |  |
| Corrected Total | 549001.006 | 44 |  |  |  |
| a. R Squared = .883 (Adjusted R Squared = .860) | | | | | |

| **Tests of Between-Subjects Effects** | | | | | |
| --- | --- | --- | --- | --- | --- |
| Dependent Variable:TT4 | | | | | |
| Source | Type III Sum of Squares | df | Mean Square | F | Sig. |
| Corrected Model | 891.260^a^ | 7 | 127.323 | 91.640 | .000 |
| Intercept | 2676.289 | 1 | 2676.289 | 1926.252 | .000 |
| DBP | 16.006 | 3 | 5.335 | 3.840 | .017 |
| TG | 825.888 | 1 | 825.888 | 594.431 | .000 |
| DBP * TG | 71.284 | 3 | 23.761 | 17.102 | .000 |
| Error | 51.407 | 37 | 1.389 |  |  |
| Total | 3634.616 | 45 |  |  |  |
| Corrected Total | 942.667 | 44 |  |  |  |
| a. R Squared = .945 (Adjusted R Squared = .935) | | | | | |

**Figure 5：**

| **Tests of Between-Subjects Effects** | | | | | |
| --- | --- | --- | --- | --- | --- |
| Dependent Variable: ROS | | | | | |
| Source | Type III Sum of Squares | df | Mean Square | F | Sig. |
| Corrected Model | 183716.759^a^ | 7 | 26245.251 | 17.527 | .000 |
| Intercept | 1.574E7 | 1 | 1.574E7 | 10508.588 | .000 |
| DBP | 148047.303 | 3 | 49349.101 | 32.955 | .000 |
| TG | 33058.822 | 1 | 33058.822 | 22.077 | .000 |
| DBP * TG | 2069.472 | 3 | 689.824 | .461 | .711 |
| Error | 56903.067 | 38 | 1497.449 |  |  |
| Total | 1.621E7 | 46 |  |  |  |
| Corrected Total | 240619.826 | 45 |  |  |  |
| a. R Squared = .764 (Adjusted R Squared = .720) | | | | | |

| **Tests of Between-Subjects Effects** | | | | | |
| --- | --- | --- | --- | --- | --- |
| Dependent Variable: 8-OHdG | | | | | |
| Source | Type III Sum of Squares | df | Mean Square | F | Sig. |
| Corrected Model | 1.102E6 | 7 | 157438.704 | 44.710 | .000 |
| Intercept | 1.093E7 | 1 | 1.093E7 | 3102.610 | .000 |
| DBP | 411040.566 | 3 | 137013.522 | 38.910 | .000 |
| TG | 450309.675 | 1 | 450309.675 | 127.881 | .000 |
| DBP * TG | 121947.684 | 3 | 40649.228 | 11.544 | .000 |
| Error | 133810.590 | 38 | 3521.331 |  |  |
| Total | 1.269E7 | 46 |  |  |  |
| Corrected Total | 1235881.518 | 45 |  |  |  |
